# DNA-Templated Fluorescent Nanoclusters for Metal Ions Detection

**DOI:** 10.3390/molecules24224189

**Published:** 2019-11-19

**Authors:** Chunxia Song, Jingyuan Xu, Ying Chen, Liangliang Zhang, Ying Lu, Zhihe Qing

**Affiliations:** 1 Department of Applied Chemistry, School of Science, Anhui Agricultural University, Hefei 230036, China; songchunxia@ahau.edu.cn (C.S.); cheny@ahau.edu.cn (Y.C.); zl18720589@ahau.edu.cn (L.Z.); luy@ahau.edu.cn (Y.L.); 2 Hunan Provincial Engineering Research Center for Food Processing of Aquatic Biotic Resources, School of Chemistry and Food Engineering, Changsha University of Science and Technology, Changsha 410114, China; xjy060612@163.com

**Keywords:** DNA-template, fluorescence, nanoclusters, metal ions, detection

## Abstract

DNA-templated fluorescent nanoclusters (NCs) have attracted increasing research interest on account of their prominent features, such as DNA sequence-dependent fluorescence, easy functionalization, wide availability, water solubility, and excellent biocompatibility. Coupling DNA templates with complementary DNA, aptamers, G-quadruplex, and so on has generated a large number of sensors. Additionally, the preparation and applications of DNA-templated fluorescent NCs in these sensing have been widely studied. This review firstly focuses on the properties of DNA-templated fluorescent NCs, and the synthesis of DNA-templated fluorescent NCs with different metals is then discussed. In the third part, we mainly introduce the applications of DNA-templated fluorescent NCs for sensing metal ions. At last, we further discuss the future perspectives of DNA-templated fluorescent NCs in the synthesis and sensing metal ions in the environmental and biological fields.

## 1. Introduction

Over the past decade, fluorescent nanoclusters (NCs), especially fluorescent gold nanoclusters (F-AuNCs), fluorescent silver nanoclusters (F-AgNCs), and fluorescent copper nanoclusters (F-CuNCs) have been developed as a very active and attractive research field [1,2,3,4,5,6,7]. Fluorescent NCs are the well-defined sub-nanometer size structures that are formed by a few to a hundred metal atoms with high emission. Current fluorescent materials that are used in sensing applications mostly include organic fluorescent dyes, up-conversion nanomaterials, quantum dots, fluorescent proteins, et al. The easy photobleaching and small Stokes shifts of organic fluorescent dyes restrict their applications in complex samples [8,9]. Most of quantum dots and up-conversion nanomaterials are physically large-size and potentially toxic, which limit their practical applications in biochemical analysis [10,11]. As for fluorescent proteins, they can be easily denatured, which makes them unsuitable for in vitro applications. When compared with these fluorescent materials, few-atom NCs exhibit excellent properties, such as large Stokes shift, high fluorescence, easy functionalization, wide availability, water solubility, and excellent biocompatibility, which make NCs an admirable fluorescent material that is used widely in the sensing field [12,13]. In 2004, it was initiatively reported [14] that fluorescent NCs could be synthesized with DNA as template in aqueous solutions. Subsequently, polymers template, such as proteins [15,16], peptides [17,18], et al. have been successfully applied. This review is focused on DNA-templated fluorescent NCs.

**Figure 1:**
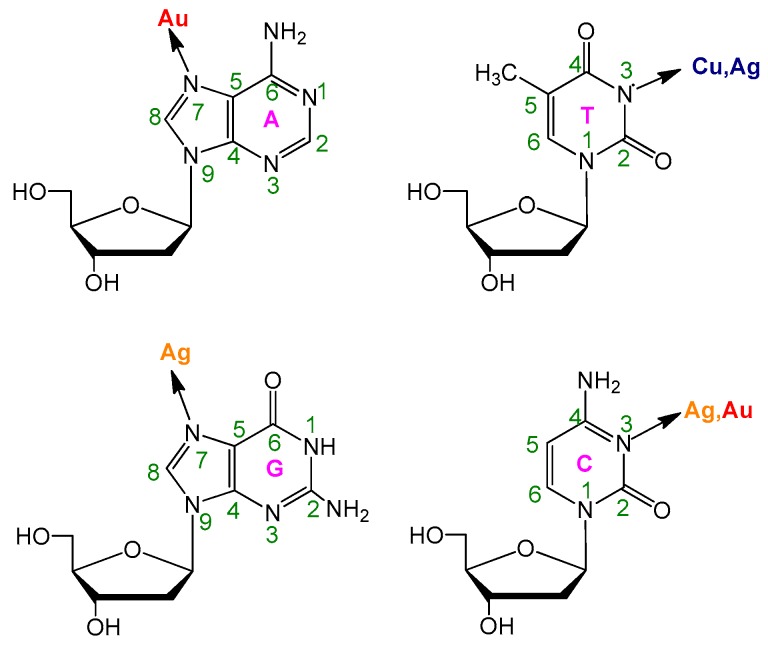
The main DNA nucleoside metal binding sites. Reprinted with permission from Ref. [5]. ©2014 American Chemical Society.

DNA is a kind of well template for fluorescent NCs synthesis and for developing sensors in metal ions detection due to its unique structure, programmable size, and pre-designable architecture when compared with other polymers [19,20]. Firstly, DNA is long-chain polymer that is composed of repeating nucleotide arrangements that are linked to each other by the 3′, 5′-phosphodiester bond. Additionally, each nucleotide consists of one nitrogenated nucleobase, one deoxyribose, and one phosphate. All of the nitrogenated nucleobases and the DNA backbone composed of deoxyriboses/phosphates are negatively charged [9]. Such a unique chemical composition of DNA provides various binding sites for metal ions through electrostatic or coordination interactions under the physiological pH condition. As shown in Figure 1 [5], each nucleotide has a metal-binding site (the N7 atoms of adenine (A) and guanine (G), the N3 atom of cytosine (T), and thymine (C)) [21]. For example, the N3 position of T and C has strong and specific interaction with metal ions Cu^2+^ and Ag^+^, respectively [22,23]. Generally, the first step of fluorescent NCs generation is the binding of metal ions onto DNA. Subsequently, these interactions among DNA and metal ions can form various inorganic nanomaterials after a further addition of reducing agents. Thus, the rich chemical functionality of DNA structure make it bind various metal ions and subsequently produce various nanomaterials of interest [21,24]. Secondly, the length of per nucleotide is 0.34 nm, and the DNA structures and nanomaterials both meet at the same size level. Thirdly, the DNA hybridization (via base-pairing of A to T and G to C) can form dsDNA structures, loop/hairpin configurations of ssDNA, and so on for a variety of nanostructures formation. Fourthly, polymerase chain reaction technology can readily synthesize and amplify high quality DNA sequences at a low price through automated methods. Fifthly, the existence of many unique and highly specific enzymes allows for the scientist to mold and manipulate DNA structures [25,26,27]. Finally, DNA aptamers can not only hybridize with complementary nucleic acids, but also selectively bind with a large number of analytes. Hence, the researchers can design DNA sequences that combine molecular recognition and advantages of DNA-templated NCs to develop new sensing strategies [28,29].

Heavy metal ions (copper (II) (Cu^2+^), mercury (II) (Hg^2+^), lead (II) (Pb^2+^), et al.,), present significant hazards to the environment and human health [30,31,32,33,34,35,36], and their pollution is getting increasingly serious due to the increase of industrial emissions, mining, and sewage irrigation in modern society [31,33,36]. Therefore, the sensitive and convenient monitoring of these heavy metal ions with low cost is urgent due to their dose dependent toxic effects. The researchers have developed various methods for metal ions monitoring today [35,36,37,38,39]. Among them, the fluorescent methods are well effective, because they possess some excellent properties, such as high sensitivity, easy preparation, and low cost [31,35,39]. Particularly, DNA-templated fluorescent NCs have been widely applied to analyze a variety of metal ions, including Cu^2+^, Hg^2+^, Pb^2+^, et al. This is a rapidly developing area with increased publications in recent years. A review on DNA-templated fluorescent NCs for metal ions sensing is extremely necessary.

In the first part, this review introduces the features of DNA-templated fluorescent NCs. Secondly, the synthesis of DNA-templated fluorescent NCs has been described. Thirdly, this review mainly introduces the application of DNA-templated fluorescent NCs for metal ions monitoring. Finally, this review trends the future prospects of DNA-templated fluorescent NCs in the synthesis and their sensing applications in environmental and biological fields. DNA-templated fluorescent NCs in this review mainly contain F-AuNCs, F-AgNCs, and F-CuNCs. Overall, we expect that this review will spark more interest in research and boost this active domain, in which DNA-templated fluorescent NCs can bring significant advantages for broader applications.

## 2. Synthesis of DNA-Templated Fluorescent Nanoclusters

**Figure 2:**
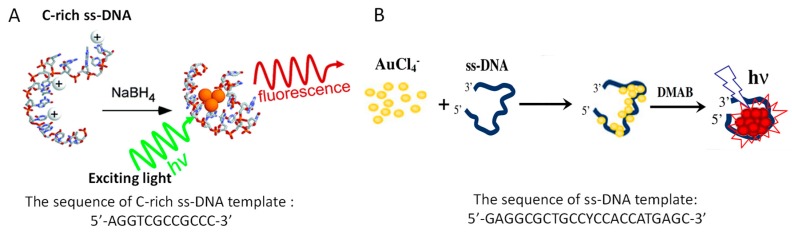
Schematic illustration of (**A**) DNA-templated AgNCs. Reprinted with permission from Ref. [14]. T Copyright 2004, American Chemical Society. (**B**) DNA-templated AuNCs. Reprinted with permission from Ref. [7]. Copyright 2012, Springer.

For analysis development, reliable sensors need to produce NCs with stable fluorescence. The fluorescence features of NCs are highly decided by their sizes, templates, solvents, synthetic strategies, et al. [40,41,42,43,44,45]. Fluorescent NCs tend to aggregate due to their high surface energy and small size, so stabilizers or matrices are needed to block NCs. In 2002, the group of Dickson [46] made the initial discovery that dendrimers were used as a template, and later they found that F-AgNCs could be synthesized with C-rich DNA as template (Figure 2A) [14]. That was because Ag^+^ could specifically coordinate with N3 position of C, which was later used to develop sensors for Ag^+^ detection [22,23]. This finding firstly connected the fluorescent NCs and DNA. The size of C-rich DNA-templated F-AgNCs is small (2.5 nm hydrodynamic radius) by consisting of several Ag atoms; interestingly, the photophysical properties of F-AgNCs can be tuned by programming different C-rich DNA sequences, which resulted in the variation of emission wavelength from blue (485 nm) to green (520 nm), yellow (572 nm), red (620 nm), and near-IR (705 nm) [47].

In the synthesis of DNA-templated F-AgNCs, C-rich DNA controls the reduced Ag ion (Ag(0)) to be aggregated on the DNA template. The reactions are mostly carried out in neutral pH, where the N3 nitrogen of C does not bear a proton; so, it is very easy for metal binding to occur. However, the N3 position of T bears a proton in neutral pH, so the synthesis of polyT-templated F-AgNCs was only reported at pH 11 [48]. As for A and G nucleosides, the N7 positions are high-affinity binding sites for metal ions, in which other nitrogens on the purine rings can promote metal coordination, so it is difficult to control reduction and form F-AgNCs on them [49,50].

Besides C-rich ssDNA, the researchers have tested a large number of DNA sequences. For example, Shao group [51] studied the impact of basic sites in dsDNA to investigate the effects of DNA-base stacking. They found that, by interacting with the G base, the excited state of DNA-templated F-AgNCs was stabilized [52]. Furthermore, mismatched dsDNA [53] and dsDNA with gaps [54] have been studied for the synthesis of DNA-templated F-AgNCs. Qu group [55] has reported that triplex DNA also supported the formation of DNA-templated F-AgNCs. In addition to the traditional “one DNA-one AgNCs” scheme, multiple DNA strands can also stabilize F-AgNCs [56]. Generally, the DNAs with the length of no less than 12-mer can support the synthesis of F-AgNCs [57]. Interestingly, although the single-C base cannot support the synthesis in water buffer, it can be used to synthesis F-AgNCs in ethanol successfully [58]. Furthermore, G-rich DNA/RNA and G-quadruplex DNA can also be applied as a template to produce F-AgNCs [59,60].

DNA-templated F-AgNCs are highly susceptible to photobleaching, and, in order to alleviate this problem, the scientists sought other suitable metal for fluorescent NCs synthesis. In 2012, the synthesis of DNA-templated F-AuNCs was reported by Shao group (Figure 2B) [7,61]. They used the sequence 5′-GAGGCGCTGCCYCCACCATGAGC-3′ as the template and dimethylamine borane (DMAB) as the reducing agent, and F-AuNCs of 5 nm was obtained at pH 7.0, with excitation and emission bands at 467 and 725 nm, respectively [7,62]. Liu group used 30-mer DNA as template and citrate as reducing agent with blue emitters of F-AuNCs being obtained. A-30 DNA could produce fluorescence at neutral pH (the ratio of A and HAuCl_4_ was 1:1), while as for the C-30 DNA, low pH was optimally in the case of excess DNA [61]. When NaBH_4_ was used as reducing agent, only large Au nanoparticles without fluorescence were obtained due to its strong reducing ability. The stoichiometric requirements, reductant, and pH might be related to the coordination between Au and bases, and then affected the formation of F-AuNCs.

**Figure 3:**
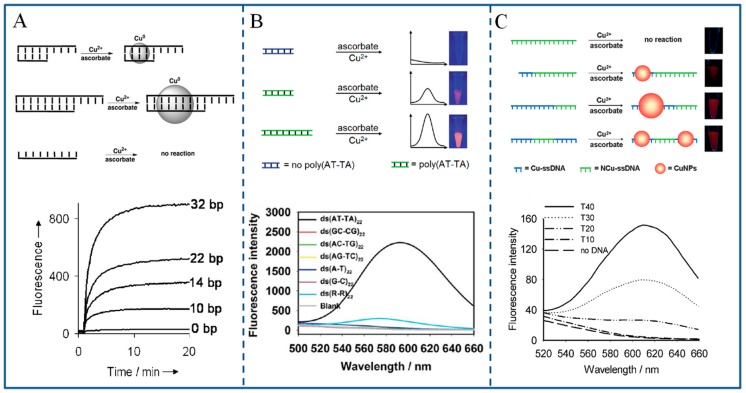
Schematic illustration of fluorescent CuNCs templated by various DNA: (**A**) dsDNA. Reprinted with permission from Ref. [63]. Copyright 2010, Wiley-VCH. (**B**) poly(AT-TA) dsDNA. The green dsDNA is the poly (AT-TA) sequence, while the blue dsDNA represents other sequences. Reprinted with permission from Ref. [65]. Copyright 2014, Royal Society of Chemistry. (**C**) polyT-DNA. The blue ssDNA is the polyT sequence, while the green ssDNA represents other sequences. Reprinted with permission from Ref. [67]. Copyright 2013, Wiley-VCH. In each case, the fluorescence intensity is proportional to the length of the template.

The superior properties of DNA-templated F-CuNCs are their simple synthesis, tunable fluorescence, and low-cost when compared to DNA-templated F-AgNCs/F-AuNCs. In 2010, Morhir et al. (Figure 3A) [63] originally discovered that F-CuNCs could be synthesized with dsDNA as template, while ssDNA did not work. Subsequently, it was found by Song et al. [64] and us [65] that the poly(AT-TA) dsDNA sequences could promote the synthesis of F-CuNCs efficiently (Figure 3B). Later, Liu et al. [66] and our group [67] found that F-CuNCs could be selectively synthesized in the presence of polyT-DNA (Figure 3C), while ssDNA with other bases did not work. In 2018, Li et al. [68] efficiently synthesized F-CuNCs with the reticular DNA as template. In general, the main steps for the formation of DNA-templated F-CuNCs include [63,66,67]: (1) Cu(II) is reduced to Cu(I) by reducing agent; (2) Cu(I) is disproportionated to Cu(0); and, (3) the aggregation of Cu(0) on the DNA templates, thus F-CuNCs are produced. Figure 3 shows the schematic illustration and fluorescence signal of various DNA-templated F-CuNCs. It is demonstrated that the fluorescence emission of DNA-templated F-CuNCs is mainly dependent on the length of DNA template. With excitation of 340 nm, DNA-templated CuNCs emit at 600 nm. The size of F-CuNCs is a few nanometers and in proportion to the length of DNA templates.

Thus, the synthesis of DNA-templated fluorescent NCs is very convenient and simple. In a typical process, metal ion (Au^+^, Ag^+^ or Cu^2+^) and DNA are mixed in water or a certain buffer with a certain ratio, to which freshly prepared reducing agent (NaBH_4_, sodium ascorbate, et al.,) is added. The DNA-templated NCs are usually only obtained for a few minutes or hours, and their emission can be regulated from green to red through the adjustment of DNA sequences and reducing agents.

Due to the extensive research and application of DNA-templated fluorescent NCs, their fluorescence properties have been extensively studied in recent years [1,5,8]. The DNA template shows significant effects on the fluorescence characteristics of NCs. For instance, the dsDNA-templated F-CuNCs show high fluorescence at the range of 587–600 nm [63]. Otherwise, the maximum fluorescence emission of polyT-templated F-CuNCs is about 615 nm [3,4,67]. In addition, the fluorescence characteristics of DNA-templated NCs are also dramatically influenced by the reducing agent. For example, DNA-templated F-AuNCs can exhibit different emission colors due to a different reducing agent [7].

## 3. DNA-Templated Fluorescent Nanoclusters for Sensing Metal Ions

In recent years, DNA-templated fluorescent NCs have been widely applied in the fields of sensing, bioimaging, therapy, catalysis, and so on [3,4,5,43,44,69]. Additionally, one very important application is metal ions detection (as summarized in Table 1) [35,39]. In general, the mechanism of DNA-templated fluorescent NCs based metal ions detection can be classified into three modes: fluorescence turn-on, ratiometric fluorescence, and fluorescence turn-off. Most of the turn-off systems are based on the fact that the fluorescence of DNA-templated NCs is susceptible to the detection medium. Otherwise, fluorescence turn-on/ratiometric systems are more sensitive. Therefore, we will focus on the application of DNA-templated fluorescent NCs in different modes for metal ions sensing.

### 3.1. Copper Ions

**Figure 4:**
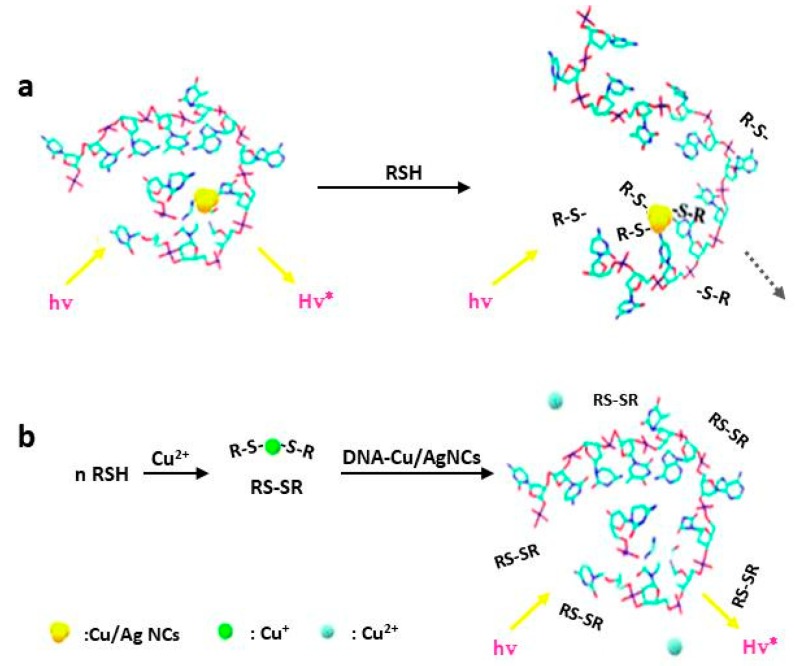
Schematic representation of turn-on Cu^2+^ sensing strategy that based on Cu^2+^ induced fluorescence recovery of DNA-templated F-Cu/AgNCs. Reprinted with permission from Ref. [74]. Copyright 2010, American Chemical Society.

As an essential trace element, copper is distributed widely in tissues and a component of hemocyanin in the human body [70,71], but excessive exposure to Cu^2+^ can interfere with cellular metabolism and lead to some neurodegenerative diseases, for example Wilson disease, Alzheimer′ Silent disease, amyotrophic lateral sclerosis, and Menkes syndrome [72]. In metal ions, the toxicity of Cu^2+^ is slightly less than Hg^2+^ in drinking water. Based on the discovery that the introduction of Cu^2+^ to the system of DNA-templated F-AgNCs could form DNA-templated Cu/Ag alloy NCs with the emission of the F-AgNCs to be −4.5-fold enhanced, a sensitive turn-on detection probe was developed by Chang group [73] for Cu^2+^ sensing (the detection limit (LOD): 10 nM). The fluorescence intensity of this probe increased along with the increasing concentration of Cu^2+^, and the practical application for Cu^2+^ measurement was verified in pond water and Montana soil. Not long after, based on the discovery that the fluorescence of DNA-templated F-Cu/AgNCs could be quenched by mercaptopropionic acid, and the introduction of Cu^2+^ could recover the fluorescence; Chang group (Figure 4) [74] has developed another sensitive method for Cu^2+^ sensing (LOD: 2.7 nM), and this method showed high specificity as the target signal was at its lowest 2300 times over other metal ions. In addition, the practical application of this method was proven through the pond water and Montana soil analyses. In 2011, Ye group [75] synthesized F-AgNCs by utilizing a C-rich ssDNA with the sequence 5′-ATCCTCCCACCGGGCCTCCCACCATAAAAACCCTTAATCCCC-3′ as the template. Interestingly, they found that Cu^2+^ could efficiently quench the fluorescence of F-AgNCs. Even though the mechanism of quenching was not very clear, they developed a turn-off Cu^2+^ detection method (LOD: 10 nM).

**Figure 5:**
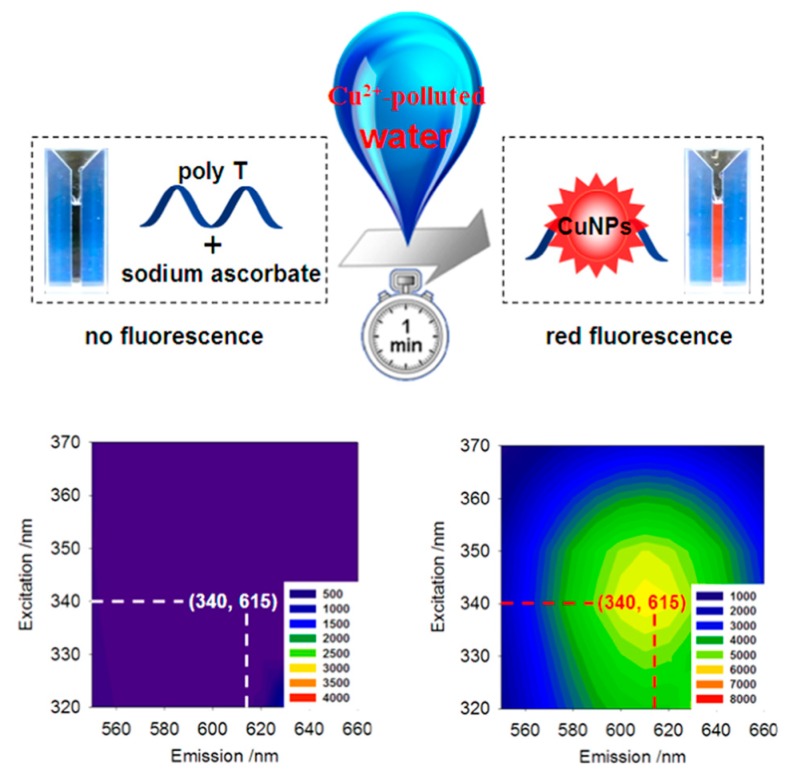
Schematic diagram of zero-background Cu^2+^ sensing/toxicides screening based on polyT-templated fluorescent copper nanoclusters (F-CuNCs) and excitation/emission spectra of polyT-templated F-CuNCs. Reprinted with permission from Ref. [31]. Copyright 2016, Elsevier B.V.

After our group [66] found that polyT-DNA could be selectively used as template to synthesize DNA-templated F-CuNCs, we [30] have constructed a turn-on and portable sensor for Cu^2+^ detection by utilizing microwell-printed hydrogel that was functionalized by polyT-DNA. Cu^2+^ could induce the in-situ formation of F-CuNCs in the hydrogel and this phenomenon could be directly observed by the naked eye under ultraviolet light. Based on the fluorescence intensity, the Cu^2+^ concentration has been determined (LOD:20 μM). Attractively, a portable microarray could be obtained by printing on the hydrogel with simply equipped instruments and used in certain remote areas. Later, in view of the in-situ synthesis of polyT-templated F-CuNCs, our group (Figure 5) [31] constructed an ultrafast fluorescent method for zero-background Cu^2+^ sensing and screening of its toxicides. The concentration of Cu^2+^ in the contaminated water was proportional to the fluorescence intensity of the polyT-templated F-CuNCs, which could be simply detected by measuring fluorescence emission. Most importantly, the production of the fluorescent signal was very fast (in just 1 min.), with high selectivity being attributed to the in situ formation. The practical use of this method has been proven by detecting Cu^2+^ from contaminated river/tap water samples, and showed good performance. Moreover, by directly analyzing the influence of different molecules on Cu^2+^, the use of this method in the screening of Cu^2+^ toxicides was verified, which showed great potential in medical therapy and the treatment of Cu^2+^-based sewage.

### 3.2. Mercury Ions

**Figure 6:**
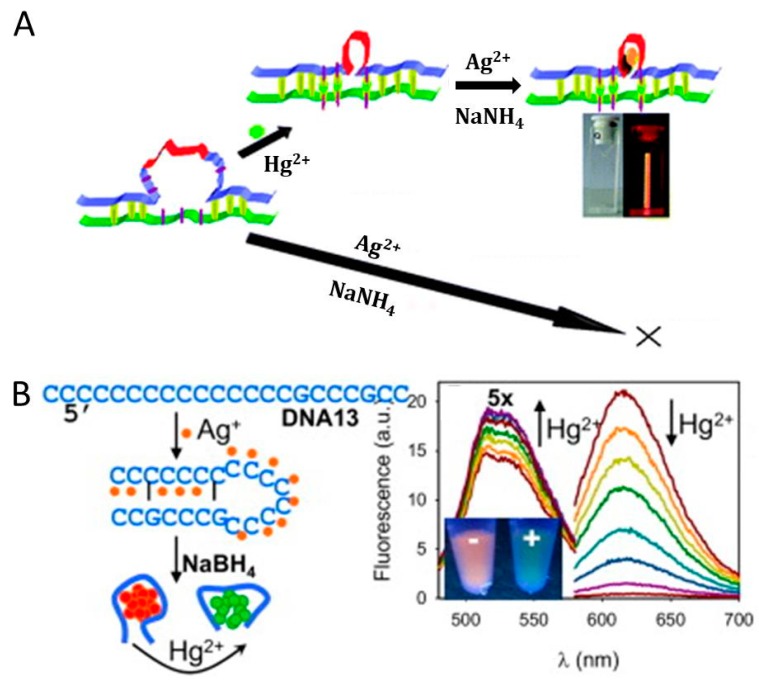
Schematic representation for (**A**) A turn-on Hg^2+^ sensor that based on DNA-templated F-AgNCs. Reprinted with permission from Ref. [78]. Copyright 2011, Royal Society of Chemistry. (**B**) A ratiometric Hg^2+^ sensor that based on dual emissive DNA-templated F-AgNCs. Reprinted with permission from Ref. [80]. Copyright 2013, Elsevier B.V.

As the most common toxic heavy metal ions existed in the environment, the accumulation of Hg^2+^ in the human body can bring about severe damage to the immune, endocrine, and nervous system, as Hg^2+^ can accumulate in the body and be easily absorbed by the skin as well as the respiratory and digestive tract. Furthermore, Hg^2+^ can bind to DNA due to the high affinity and damage nervous/brain system [32,35,36]. Wang group [76] have found that the introduction of Hg^2+^ to the DNA-templated F-AgNCs could form a non-fluorescent complex between Hg^2+^ and DNA-templated F-AgNCs, resulting in the impairing of the interaction between the F-AgNCs and DNA template with fluorescence quenched. By utilizing this discovery, they reported a sensitive turn-off Hg^2+^ detection method (LOD: 5 nM) with high selectivity. The linear Stern-Volmer plot and unchanged lifetime of fluorescence indicated that static quenching was the mechanism of this Hg^2+^ induced quenching. Lan et al. [77] reported another turn-off Hg^2+^ detection method (LOD: 0.9 nM), in which the DNA-templated F-AgNCs was prepared by using a molecular beacon (5′-CCCTTCCTTCCTTCCAACCAACCC-3′) and the quantum yield of this DNA-templated F-AgNCs probe (at 608 nm) was as high as 61% with high stability towards exonuclease I^-^, thiols ^-^, and Cl^-^. Deng et al. (Figure 6A) [78] developed a turn-on method for Hg^2+^ sensing that was based on the fact that Hg^2+^ could stabilize T-T mismatch. In this case, the DNA duplexes as capping scaffolds (hybridized by one ssDNA with C-loop inside and another DNA) had some T-T base mismatches on the loop. T-T formation that was mediated by Hg^2+^ could strength the DNA duplexes, resulting in the high yield of DNA-templated F-AgNCs, thus a turn-on strategy for Hg^2+^ sensing has been created (LOD: 10 nM in water). In 2013, Yin et al. [79] designed another turn-on Hg^2+^ fluorescence sensor (LOD was 0.08 nM) that utilized T-Hg^2+^-T complexes and DNA molecular machine-based F-AgNCs. Hg^2+^ could trigger machine-like operations of DNA with a mass of DNA produced, and the “product” DNA could be used to stabilize F-AgNCs. In 2013, Liu et al. (Figure 6B) [80] proposed a ratiometric and visual Hg^2+^ sensor that was based on dual emissive DNA-templated F-AgNCs with a LOD of 4 nM. The introduction of Ag^+^ to a C-rich ssDNA could stabilize the C-Ag^+^-C base pair [81] and one hairpin structure formed. Additionally, the gained hairpin DNA was employed for the synthesis of orange emission F-AgNCs. Interestingly, in the presence of Hg^2+^, the emission was changed to green. By just varying the DNA structure, a ratiometric method for sensitive Hg^2+^ sensing was achieved. In addition, they successfully immobilized this sensor in a monolithic hydrogel matrix for easy manipulation and device incorporation.

**Figure 7:**
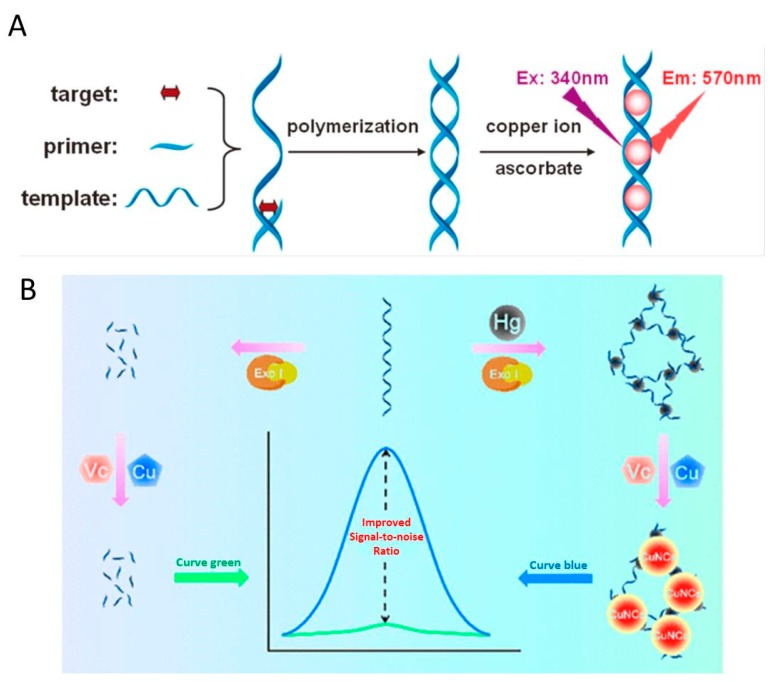
Schematic representation for (**A**) A turn-on strategy for polymerization-mediated Hg^2+^ detection based on dsDNA-templated F-CuNCs. Reprinted with permission from Ref. [42]. Copyright 2014, Royal Society of Chemistry; (**B**) A high signal-to-noise ratio strategy for Hg^2+^ sensing that based on rigid reticular DNA templated F-CuNCs. Vc is the abbreviation of ascorbate, and Exo I is the abbreviation of exonuclease I. Reprinted with permission from Ref. [68]. Copyright 2018, American Chemical Society.

Based on dsDNA-templated F-CuNCs and T-T formation that mediated by Hg^2+^, our group (Figure 7A) [42] have proposed a facile strategy for Hg^2+^ sensing. The introduction of Hg^2+^ could promote the hybridization of the primer and template DNA, resulting in the primer-extension reaction and the produced dsDNA could be used for the formation of F-CuNCs. This turn-on method showed good selectivity in Hg^2+^ detection and has been applied in nucleic acid and polymerase detection. Based on polyT-templated F-CuNCs, Wang et al. [82] developed another turn-on strategy for Hg^2+^ sensing. This label-free strategy has been used to detect Hg^2+^ on the basis that Hg^2+^ could block the cysteine induced quenching of polyT-templated F-CuNCs. The fluorescent probe (T30-DNA templated F-CuNCs) could be produced within 5 min. Cysteine could form the coordination complex with Cu in the T30-templated F-CuNCs through the Cu-S metal-ligand bond and then effectively quenched the fluorescence. However, on account of the strong binding between cysteine and Hg^2+^, the introduction of Hg^2+^ could form a more stable Hg-S bond, which made cysteine depart from T30-templated F-CuNCs with recovered fluorescence. This strategy achieved good sensitivity (LOD: 0.1 nM) and excellent selectivity. Moreover, the practical detection of Hg^2+^ has been successfully achieved in the lake water samples. In 2018, Li et al. (Figure 7B) [68] developed a high signal-to-noise ratio Hg^2+^ strategy that was based on reticular DNA-templated F-CuNCs. The introduction of Hg^2+^ to the polyT-DNA could form T-Hg^2+^-T nodes, and rigid reticular DNA was formed in the end, which could be used to synthesize F-CuNCs with enhanced fluorescence. The excessive polyT-DNA was digested by exonuclease I, with reduced background and high signal-to-noise ratio being gained.

### 3.3. Lead Ions

**Figure 8:**
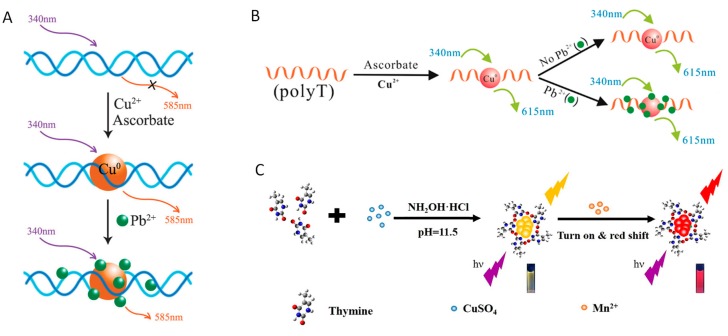
Schematic representation for (**A**) Turn-off sensing of Hg^2+^ based on dsDNA-templated F-CuNCs. Reprinted with permission from Ref. [85]. Copyright 2012, Royal Society of Chemistry; (**B**) PolyT-templated F-CuNCs based turn-off sensing of Hg^2+^. Reprinted with permission from Ref. [86]. Copyright 2014, Japan Society for Analytical Chemistry; (**C**) Turn-off detection of Mn^2+^ using single T-templated F-CuNCs. Reprinted with permission from Ref. [90]. Copyright 2017, Royal Society of Chemistry.

Pb^2+^ is a serious toxic heavy metal ion that is widely distributed in drinking water. High level of Pb^2+^ can cause serious damage to human body, including impaired growth, decreased intelligence, irritability muscle paralysis, and kidney disease [35,36,83,84]. The Zeng group (Figure 8A) [85] has discovered that Pb^2+^ could selectively quench the fluorescence of dsDNA-templated F-CuNCs, owing to the metallophilic interactions at the surface of F-CuNCs through 5d10(Pb^2+^)–3d10(Cu^2+^) reaction. Based on this fact, a turn-off method has been achieved with good sensitivity (LOD: 5 nM), and the practical application of this method has been verified for Pb^2+^ detection in river water and human urine samples. By a similar mechanism, another turn-off strategy (Figure 8B) [86] that based on polyT-templated F-CuNCs was proven to effectively detect Pb^2+^ with high sensitivity (the LOD was 0.4 nM) and good selectivity. Besides, the practical assay of Pb^2+^ has been successfully achieved for the tap water samples.

### 3.4. Other Ions

Manganese (Mn) is one of the essential trace elements in the human body, which constitutes several important physiologically active enzymes in the body [87,88]. Mn^2+^ deficiency can lead to hypercholesterolaemia and delay blood coagulation. However, excessive intake of Mn^2+^ might cause adverse neurological effects and manganism [89]. Interestingly, in 2013, Han et al. (Figure 8C) [90] found that besides poly-T DNAs, single-T base could also be used as the template for the formation of F-CuNCs (the maximum excitation wavelength: 354 nm; fluorescence emission peaks: 561 nm), and the introduction of Mn^2+^ could make the emission red shifted. Based on these facts, a turn-on fluorescent method for Mn^2+^ monitoring has been established with a high sensitivity (LOD: 10 μM) and good selectivity.

## 4. Future Prospects

The applications of DNA-templated fluorescent NCs in sensing metal ions have been summarized in this review. In recent years, the robustness and versatility of DNA-templated fluorescent NCs have made them promising candidates for detecting various metal ions. It was shown that there are some convincing features of the DNA-templated fluorescent NCs: (1) Excellent characteristics of fluorescence: the large MegaStokes shift of the DNA-templated fluorescent NCs makes them potentially useful in complex matrices of actual samples; (2) Label-free: there is no need for labeling, without complex synthesis and operation procedures; (3) Rapid: the interaction between target and DNA templates or NCs is the basis of most metal ions detection, which is rapid and finished in a few minutes; (4) Environmental-benign: as compared with quantum dots and other nanomaterials, DNA-templated NCs are less bio-toxic; and, (5) Sensitive and versatile: by associating the DNA signal amplification with target-recognition capability [27], the DNA-templated fluorescent NCs-based assays are easily and simply designed to obtain high sensitivity and versatility.

Here, we also discuss personal views from the authors regarding a few future improvements and novel applications: (1) The basic mechanism study: it is necessary to systematically establish the relationship between the emission of the DNA-templated fluorescent NCs and their sequences of DNA templates. The research needs to screen a great many of DNAs with various length and sequences. Besides, it is urgent to understand the structure of the DNA-NCs complex in order to design the DNA sequences rationally with a highly successful rate; (2) Long-term photostability: DNA-templated fluorescent NCs are usually unstable and the fluorescence is continuously reduced since formation. More stable DNA-templated fluorescent NCs should be achieved through the rational design of the DNA sequence and length, and the experimental conditions need to be carefully controlled; and, (3) Novel analytical strategies: application and exploration of novel analytical strategies may exhibit great prominence. Some attempts, such as electrochemical assay [92,93], plasma mass spectrometer (ICP-MS) assay [94], surface plasmon resonance (SPR) assay [95], chemiluminescent assay [96,97], electrochemiluminescent assay [98,99], real-time/on-site colorimetric assay [100,101], surface-enhanced Raman scattering (SERS) assay [102], et al. have brought interesting effects. In summary, as shown in this review, the metal ions assay based on DNA-templated fluorescent NCs has opened a new way and might hold great practical potential for sensing in environmental and biological fields.

## Figures and Tables

**Table 1:** Summary of DNA-templated nanoclusters that used for metal ions detection [30,31,42,62,68,73,74,75,76,77,78,80,82,85,86,90,91].

Nanomaterial Used	Analytes	Strategy	LOD	Linear Range (nM)	Ref.
PolyT-templated F-CuNCs	Cu^2+^	Turn-on	20 µM	20 µM–10 mM	[30]
PolyT-templated F-CuNCs	Cu^2+^	Turn-on	5.6 μM	15–35 µM	[31]
DNA-templated F-AgNCs	Cu^2+^	Turn-on	8 nM	25–250 nM	[73]
DNA-templated F-AgNCs	Cu^2+^	Turn-on	2.7 nM	5–200 nM	[74]
DNA-templated F-AgNCs	Cu^2+^	Turn-off	10 nM	10–1000 nM	[75]
dsDNA-templated F-CuNCs	Hg^2+^	Turn-on	-	-	[42]
PolyT-templated F-CuNCs	Hg^2+^	Turn-on	-	-	[62]
PolyT-templated F-CuNCs	Hg^2+^	Turn-on	16 pM	50 pM–500 μM	[68]
DNA-templated F-AgNCs	Hg^2+^	Turn-off	5 nM	5–1500 nM	[76]
DNA-templated F-AgNCs	Hg^2+^	Turn-off	0.9 nM	2.5–50 nM	[77]
DNA-templated F-AgNCs	Hg^2+^	Turn-on	10 nM	10–300 nM	[78]
DNA-templated F-AgNCs	Hg^2+^	Turn-off	4 nM	10–200 nM	[80]
PolyT-templated F-CuNCs	Hg^2+^	Turn-on	0.1 nM	0.5–30 nM	[82]
DNA-templated F-AgNCs	Hg^2+^	Turn-on	0.033 nM	0.1–200 nM	[91]
dsDNA-templated F-CuNCs	Pb^2+^	Turn-off	5 nM,	5–100 nM	[85]
PolyT-templated F-CuNCs	Pb^2+^	Turn-off	0.4 nM	1.0–500 nM	[86]
SingleT-templated F-CuNCs	Mn^2+^	Turn-on	10 μM.	100–250 μM	[90]

## References

[1] Qing Z., Bai A., Xing S., Zou Z., He X., Wang K., Yang R. (2019). Progress in biosensor based on DNA-templated copper nanoparticles. Biosens. Bioelectron..

[2] Zhang L., Wang E. (2014). Metal nanoclusters: New fluorescent probes for sensors and bioimaging. Nano Today.

[3] Guo Y., Cao F., Lei X., Mang L., Cheng S., Song J. (2016). Fluorescent copper nanoparticles: Recent advances in synthesis and applications for sensing metal ions. Nanoscale.

[4] Rui L., Wang C., Hu J., Su Y., Yi L. (2018). DNA-templated copper nanoparticles: Versatile platform for label-free bioassays. Trends Anal. Chem..

[5] Liu J. (2014). DNA-stabilized, fluorescent, metal nanoclusters for biosensor development. Trends Anal. Chem..

[6] Qing T., Zhang K., Qing Z., Wang X., Long C., Zhang P., Hu H., Feng B. (2019). Recent progress in copper nanocluster-based fluorescent probing: A review. Mikrochim. Acta.

[7] Liu G., Yong S., Cui Q., Fei W., Xu S. (2012). Synthesis of DNA-templated fluorescent gold nanoclusters. Gold Bull..

[8] Qing Z., Hou L., Yang L., Zhu L., Yang S., Zheng J., Yang R. (2016). A reversible nanolamp for instantaneous monitoring of cyanide based on an elsner-like reaction. Anal. Chem..

[9] Zhu L., Qing Z. (2017). Direct detection of nucleic acid with minimizing background and improving sensitivity based on a conformation-discriminating indicator. Anal. Chem..

[10] Yao J., Li L., Li P., Yang M. (2017). Quantum dots: From fluorescence to chemiluminescence, bioluminescence, electrochemiluminescence, and electrochemistry. Nanoscale.

[11] Matea C.T., Mocan T., Tabaran F., Pop T., Mosteanu O., Puia C., Iancu C., Mocan L. (2017). Quantum dots in imaging, drug delivery and sensor applications. Int. J. Nanomed..

[12] Li Y., Luo G., Qing Z. (2019). Colorimetric aminotriazole assay based on catalase deactivation-dependent longitudinal etching of gold nanorods. Microchim. Acta.

[13] Liu C., Chen W., Qing Z., Zheng J., Xiao Y., Yang S., Wang L., Li Y., Yang R. (2016). In vivo lighted fluorescence via fenton reaction: Approach for imaging of hydrogen peroxide in living systems. Anal. Chem..

[14] Petty J.T., Zheng J., Hud N.V., Dickson R.M. (2004). DNA-templated Ag nanocluster formation. J. Am. Chem. Soc..

[15] Hsu Y., Hung M., Chen Y., Wang T., Ou Y., Chen S. (2019). Identifying reducing and capping sites of protein-encapsulated gold nanoclusters. Molecules.

[16] Li Y., Jian X., Zhou S., Lu Y., Zhao C., Gao Z., Song Y.-Y. (2019). Protein shell-encapsulated Pt clusters as continuous O_2_-supplied biocoats for photodynamic therapy in hypoxic cancer cells. ACS Appl. Mater. inter..

[17] Sang F., Zhang X., Shen F. (2019). Fluorescent methionine-capped gold nanoclusters for ultra-sensitive determination of copper(II) and cobalt(II), and their use in a test strip. Mikrochim. Acta.

[18] Xu S., Li W., Zhao X., Wu T., Cui Y., Fan X., Wang W., Luo X. (2019). Ultra-highly efficient and stable fluorescent gold nanoclusters coated with screened peptides of unique sequences for effective protein and serum discrimination. Anal. Chem..

[19] Wilner O.I., Willner I. (2012). Functionalized DNA nanostructures. Chem. Rev..

[20] Zhang G., Surwade S.P., Zhou F., Liu H. (2013). DNA nanostructure meets nanofabrication. Chem. Soc. Rev..

[21] Berti L., Burley G.A. (2008). Nucleic acid and nucleotide-mediated synthesis of inorganic nanoparticles. Nat. Nanotechnol..

[22] Ono A., Torigoe H., Tanaka Y., Okamoto I. (2011). Binding of metal ions by pyrimidine base pairs in DNA duplexes. Chem. Soc. Rev..

[23] Ono A., Cao S., Togashi H., Tashiro M., Fujimoto T., Machinami T., Oda S., Miyake Y., Okamoto I., Tanaka Y. (2008). Specific interactions between silver(I) ions and cytosine–cytosine pairs in DNA duplexes. Chem. Commun..

[24] Pompa P.P., Martiradonna L., Della Torre A., Della Sala F., Manna L., De Vittorio M., Calabi F., Cingolani R., Rinaldi R. (2006). Metal-enhanced fluorescence of colloidal nanocrystals with nanoscale control. Nat. Nanotechnol..

[25] Peng Z., Liu H. (2016). Bottom-up nanofabrication using DNA nanostructures. Chem. Mater..

[26] Qing Z., Zhu L., Hou L., Zou Z., Yang S., Yang R. (2018). A dsDNA-lighted fluorophore for monitoring protein-ligand interaction through binding-mediated DNA protection. Sci. China Chem..

[27] Qing Z., Xu J., Hu J., Zheng J., He L., Zou Z., Yang S., Tan W., Yang R. (2019). In situ amplification-based imaging of RNA in living cells. Angew. Chem. Int. Ed..

[28] Yang L., Qing Z., Liu C., Tang Q., Li J., Yang S., Zheng J., Yang R., Tan W. (2016). Direct fluorescent detection of blood potassium by ion-selective formation of intermolecular G-Quadruplex and ligand binding. Anal. Chem..

[29] Zhang X., Song C., Yang K., Hong W., Lu Y., Yu P., Mao L. (2018). Photoinduced regeneration of an aptamer-based electrochemical sensor for sensitively detecting adenosine triphosphate. Anal. Chem..

[30] Qing Z., Mao Z., Qing T., He X., Zou Z., He D., Shi H., Huang J., Liu J., Wang K. (2014). Visual and portable strategy for copper(II) detection based on a striplike poly(thymine)-caged and microwell-printed hydrogel. Anal. Chem..

[31] Qing Z., Zhu L., Yang S., Cao Z., He X., Wang K., Yang R. (2016). In situ formation of fluorescent copper nanoparticles for ultrafast zero-background Cu^2+^ detection and its toxicides screening. Biosens. Bioelectron..

[32] Qing Z., Zhu L., Li X., Yang S., Zou Z., Guo J., Cao Z., Yang R. (2017). A target-lighted dsDNA-indicator for high-performance monitoring of mercury pollution and its antagonists screening. Environ. Sci. Technol..

[33] Yaita T. (2013). Trace copper(II) ions detection and removal from water using novel ligand modified composite adsorbent. Chem. Eng. J..

[34] Knecht M.R., Manish S. (2009). Bio-inspired colorimetric detection of Hg^2+^ and Pb^2+^ heavy metal ions using Au nanoparticles. Anal. Bioanal. Chem..

[35] Guo Y., Zhang L., Zhang S., Yang Y., Chen X., Zhang M. (2015). Fluorescent carbon nanoparticles for the fluorescent detection of metal ions. Biosens. Bioelectron..

[36] Guo Y., Wang Z., Qu W., Shao H., Jiang X. (2011). Colorimetric detection of mercury, lead and copper ions simultaneously using protein-functionalized gold nanoparticles. Biosens. Bioelectron..

[37] Bansod B., Kumar T., Thakur R., Rana S., Singh I. (2017). A review on various electrochemical techniques for heavy metal ions detection with different sensing platforms. Biosens. Bioelectron..

[38] Qian X., Xu Z. (2015). Fluorescence imaging of metal ions implicated in diseases. Chem. Soc. Rev..

[39] Huang J., Su X., Li Z. (2017). Metal ion detection using functional nucleic acids and nanomaterials. Biosens. Bioelectron..

[40] Chen L., Wang C., Yuan Z., Chang H. (2015). Fluorescent gold nanoclusters: Recent advances in sensing and imaging. Anal. Chem..

[41] Schaeffer N., Tan B., Dickinson C., Rosseinsky M.J., Laromaine A., Mccomb D.W., Stevens M.M., Wang Y., Petit L., Barentin C. (2008). Fluorescent or not? Size-dependent fluorescence switching for polymer-stabilized gold clusters in the 1.1–1.7 nm size range. Chem. Commun..

[42] Qing Z.H., Qing T.P., Mao Z.G., He X.X., Wang K.M., Zou Z., Shi H., He D.G. (2014). dsDNA-specific fluorescent copper nanoparticles as a “green” nano-dye for polymerization-mediated biochemical analysis. Chem. Commun..

[43] Song C., Yang X., Wang K., Wang Q., Huang J., Liu J., Liu W., Liu P. (2014). Label-free and non-enzymatic detection of DNA based on hybridization chain reaction amplification and dsDNA-templated copper nanoparticles. Anal. Chim. Acta.

[44] Song C., Hong W., Zhang X., Lu Y. (2018). Label-free and sensitive detection of ochratoxin A based on dsDNA-templated copper nanoparticles and exonuclease-catalyzed target recycling amplification. Analyst.

[45] Xu F., Shi H., He X., Wang K., He D., Guo Q., Qing Z., Yan L., Ye X., Li D. (2014). Concatemeric dsDNA-templated copper nanoparticles strategy with improved sensitivity and stability based on rolling circle replication and its application in microRNA detection. Anal. Chem..

[46] Zheng J., Dickson R.M. (2002). Individual water-soluble dendrimer-encapsulated silver nanodot fluorescence. J. Am. Chem. Soc..

[47] Richards C.I., Choi S., Hsiang J., Antoku Y., Vosch T., Bongiorno A., Tzeng Y., Dickson R.M. (2008). Oligonucleotide-stabilized Ag nanocluster fluorophores. J. Am. Chem. Soc..

[48] Sengupta B., Ritchie C.M., Buckman J.G., Johnsen K.R., Goodwin P.M., Petty J.T. (2008). Base-directed formation of fluorescent silver clusters. J. Phys. Chem. C.

[49] Navarro J.A.R., Lippert B. (1999). Molecular architecture with metal ions, nucleobases and other heterocycles. Coord. Chem. Rev..

[50] Verma S., Mishra A.K., Kumar J. (2010). The many facets of adenine: Coordination, crystal patterns, and catalysis. Acc. Chem. Res..

[51] Ma K., Shao Y., Cui Q., Wu F., Xu S., Liu G. (2012). Base-stacking-determined fluorescence emission of DNA abasic site-templated silver nanoclusters. Langmuir.

[52] Ma K., Cui Q., Liu G., Wu F., Xu S., Shao Y. (2011). DNA abasic site-directed formation of fluorescent silver nanoclusters for selective nucleobase recognition. Nanotechnology.

[53] Huang Z., Pu F., Hu D., Wang C., Ren J., Qu X. (2011). Site-specific DNA-programmed growth of fluorescent and functional silver nanoclusters. Chemistry.

[54] Cui Q., Ma K., Shao Y., Xu S., Wu F., Liu G., Teramae N., Bao H. (2012). Gap site-specific rapid formation of fluorescent silver nanoclusters for label-free DNA nucleobase recognition. Anal. Chim. Acta.

[55] Feng L., Huang Z., Ren J., Qu X. (2012). Toward site-specific, homogeneous and highly stable fluorescent silver nanoclusters fabrication on triplex DNA scaffolds. Nucleic Acids Res..

[56] Zhao T., Chen Q., Zeng C., Lan Y., Cai J., Liu J., Gao J. (2013). Multi-DNA-Ag nanoclusters: Reassembly mechanism and sensing the change of HIF in cells. J. Mater. Chem. B.

[57] Konrad K., Korbinian B. (2010). A highly charged Ag_6_^4+^ core in a DNA-encapsulated silver nanocluster. Chemistry.

[58] Yang X., Gan L., Han L., Wang E., Wang J. (2013). High-yield synthesis of silver nanoclusters protected by DNA monomers and DFT prediction of their photoluminescence properties. Angew. Chem. Int. Ed..

[59] Danielle S., Elisabeth G. (2011). Stabilization of fluorescent silver clusters by RNA homopolymers and their DNA analogs: C,G versus A,T(U) dichotomy. Chem. Commun..

[60] Ai J., Guo W., Li B., Tao L., Dan L., Wang E. (2012). DNA G-quadruplex-templated formation of the fluorescent silver nanocluster and its application to bioimaging. Talanta.

[61] Kennedy T.A., Maclean J.L., Liu J. (2012). Blue emitting gold nanoclusters templated by poly-cytosine DNA at low pH and poly-adenine DNA at neutral pH. Chem. Commun..

[62] Liu G., Shao Y., Wu F., Xu S., Peng J., Liu L. (2013). DNA-hosted fluorescent gold nanoclusters: Sequence-dependent formation. Nanotechnology.

[63] Rotaru A., Dutta S., Jentzsch E., Gothelf K., Mokhir A. (2010). Selective dsDNA-templated formation of copper nanoparticles in solution. Angew. Chem. Int. Ed..

[64] Song Q., Shi Y., He D., Xu S., Ouyang J. (2015). Sequence-dependent dsDNA-templated formation of fluorescent copper nanoparticles. Chem. Eur. J..

[65] Qing T., Qing Z., Mao Z., He X., Xu F., Wen L., He D., Shi H., Wang K. (2014). dsDNA-templated fluorescent copper nanoparticles: Poly (AT-TA)-dependent formation. RSC Adv..

[66] Liu G., Shao Y., Peng J., Dai W., Liu L., Xu S., Wu F., Wu X. (2013). Highly thymine-dependent formation of fluorescent copper nanoparticles templated by ss-DNA. Nanotechnology.

[67] Qing Z., He X., He D., Wang K., Xu F., Qing T., Yang X. (2013). Poly(thymine)-templated selective formation of fluorescent copper nanoparticles. Angew. Chem. Int. Ed..

[68] Li J., Fu W., Bao J., Wang Z., Dai Z. (2018). Fluorescence regulation of copper nanoclusters via DNA template manipulation toward design of a high signal-to-noise ratio biosensor. ACS Appl. Mater. inter..

[69] Xue H., Liu T., Zhuang Y., Wei W., Li Y., Fan W., Huang Y. (2016). Recent advances in the analytical applications of copper nanoclusters. Trends Anal. Chem..

[70] Harris E.D. (1992). Copper as a cofactor and regulator of copper, zinc superoxide dismutase. J. Nutr..

[71] Plastino J., Green E.L., Sanders-Loehr J., Klinman J.P. (1999). An unexpected role for the active site base in cofactor orientation and flexibility in the copper amine oxidase from Hansenula polymorpha. Biochemistry.

[72] Chen P., Solomon E.I. (2004). Oxygen activation by the noncoupled binuclear copper site in peptidylglycine alpha-hydroxylating monooxygenase. Reaction mechanism and role of the noncoupled nature of the active site. J. Am. Chem. Soc..

[73] Lan G.Y., Huang C.C., Chang H.T. (2010). Silver nanoclusters as fluorescent probes for selective and sensitive detection of copper ions. Chem. Commun..

[74] Su Y.T., Lan G.Y., Chen W.Y., Chang H.T. (2010). Detection of copper ions through recovery of the fluorescence of DNA-templated copper/silver nanoclusters in the presence of mercaptopropionic acid. Anal. Chem..

[75] Zhang M., Ye B.C. (2011). Label-free fluorescent detection of copper(II) using DNA-templated highly luminescent silver nanoclusters. Analyst.

[76] Guo W., Yuan J., Wang E. (2009). Oligonucleotide-stabilized Ag nanoclusters as novel fluorescence probes for the highly selective and sensitive detection of the Hg^2+^ ion. Chem. Commun..

[77] Lan G.-Y., Chen W.-Y., Chang H.-T. (2011). Control of synthesis and optical properties of DNA templated silver nanoclusters by varying DNA length and sequence. RSC Adv..

[78] Deng L., Zhou Z., Li J., Li T., Dong S. (2011). Fluorescent silver nanoclusters in hybridized DNA duplexes for the turn-on detection of Hg^2+^ ions. Chem. Commun..

[79] Yin J., He X., Jia X., Wang K., Xu F. (2013). Highly sensitive label-free fluorescent detection of Hg^2+^ ions by DNA molecular machine-based Ag nanoclusters. Analyst.

[80] MacLean J.L., Morishita K., Liu J. (2013). DNA stabilized silver nanoclusters for ratiometric and visual detection of Hg^2+^ and its immobilization in hydrogels. Biosens. Bioelectron..

[81] Ono A., Togashi H. (2004). Highly selective oligonucleotide-based sensor for mercury(II) in aqueous solutions. Angew. Chem. Int. Ed..

[82] Wang H., Chen Y., Li Y., Zhang H., Cao J. (2015). A rapid, sensitive and label-free sensor for Hg(II) ion detection based on blocking of cysteine-quenching of fluorescent poly(thymine)-templated copper nanoparticles. RSC Adv..

[83] Tipton I.H. (1968). The human body burden of lead. Arch. Environ. Health.

[84] Wasserman G., Graziano J.H., Factor-Litvak P., Md D.P., Morina M.N., Musabegovic M.A., Vrenezi M.N., Capuni-Paracka M.S., Lekic M.V., Preteni-Redjepi M.E. (1992). Independent effects of lead exposure and iron deficiency anemia on developmental outcome at age 2 years. J. Pediatr..

[85] Chen J., Liu J., Fang Z., Zeng L. (2012). Random dsDNA-templated formation of copper nanoparticles as novel fluorescence probes for label-free lead ions detection. Chem. Commun..

[86] Ou L., Li X., Liu H., Li L., Chu X. (2014). Poly(thymine)-templated fluorescent copper nanoparticles for ultrasensitive label-free detection of Pb^2+^ ion. Anal. Sci..

[87] Adams L.F., Ghiorse W.C. (1987). Characterization of extracellular Mn^2+^-oxidizing activity and isolation of an Mn^2+^-oxidizing protein from Leptothrix discophora SS-1. J. Bacteriol..

[88] Hu S., Zhang J., Tang R., Fan J., Liu H., Kang W., Lei C., Nie Z., Huang Y., Yao S. (2019). Click-type protein-DNA conjugation for Mn^2+^ imaging in living cells. Anal. Chem..

[89] Zhang J., Chen X., Yue L., Han S., Yao D., Liu H. (2018). Nitrogen doped carbon quantum dots-enhanced chemiluminescence method for the determination of Mn^2+^. Anal. Methods.

[90] Han B., Xiang R., Hou X., Yu M., Peng T., Li Y., He G. (2017). One-step rapid synthesis of single thymine-templated fluorescent copper nanoclusters for “turn on” detection of Mn^2+^. Anal. Methods.

[91] Wang G., Xu G., Zhu Y., Zhang X. (2013). A “turn-on” carbon nanotube-Ag nanoclusters fluorescent sensor for sensitive and selective detection of Hg^2+^ with cyclic amplification of exonuclease III activity. Chem. Commun..

[92] Miao P., Zhang T., Xu J., Tang Y. (2018). Electrochemical detection of miRNA combining T7 exonuclease-assisted cascade signal amplification and DNA-templated copper nanoparticles. Anal. Chem..

[93] Sheng F., Zhang X., Wang G. (2017). Novel ultrasensitive homogeneous electrochemical aptasensor based on dsDNA-templated copper nanoparticles for the detection of ractopamine. J. Mater. Chem. B.

[94] Liu R., Wang C., Xu Y., Hu J., Deng D., Lv Y. (2017). Label-free DNA assay by metal stable isotope detection. Anal. Chem..

[95] Yuan P.-X., Deng S.-Y., Zheng C.-Y., Cosnier S., Shan D. (2017). In situ formed copper nanoparticles templated by TdT-mediated DNA for enhanced SPR sensor-based DNA assay. Biosens. Bioelectron..

[96] Zhu N., Gu L., Wang J., Li X., Liang G., Zhou J., Zhang Z. (2019). Novel and sensitive chemiluminescence sensors based on 2D-MOF nanosheets for one-step detection of glucose in human urine. J. Phys. Chem. C.

[97] Yang N., Huang Y., Ding G., Fan A. (2019). In situ generation of prussian blue with potassium ferrocyanide to improve the sensitivity of chemiluminescence immunoassay using magnetic nanoparticles as label. Anal. Chem..

[98] Zhang B., Zhang F., Zhang P., Shen D., Gao X., Zou G. (2019). Ultrasensitive electrochemiluminescent sensor for MicroRNA with multinary Zn-Ag-In-S/ZnS nanocrystals as tags. Anal. Chem..

[99] Li S., Liu Z., Li J., Chen A., Chai Y., Yuan R., Zhuo Y. (2018). Enzyme-free target recycling and double-output amplification system for electrochemiluminescent assay of Mucin 1 with MoS_2_ nanoflowers as co-reaction accelerator. ACS Appl. Mater. inter..

[100] Zhuang Y.-T., Chen S., Jiang R., Yu Y., Wang J. (2019). Ultrasensitive colorimetric chromium chemosensor based on dye color switching under the Cr(VI)-stimulated Au NPs catalytic activity. Anal. Chem..

[101] Wolfe M.G., Ali M.M., Brennan J.D. (2019). Enzymatic litmus test for selective colorimetric detection of C-C single nucleotide polymorphisms. Anal. Chem..

[102] Zhou Q., Zheng J., Qing Z., Zheng M., Yang J., Yang S., Ying L., Yang R. (2016). Detection of circulating tumor DNA in human blood via DNA-mediated surface-enhanced Raman spectroscopy of single-walled carbon nanotubes. Anal. Chem..

